# Susceptibility to COVID-19 Scams: The Roles of Age, Individual Difference Measures, and Scam-Related Perceptions

**DOI:** 10.3389/fpsyg.2021.789883

**Published:** 2021-12-15

**Authors:** Julia Nolte, Yaniv Hanoch, Stacey Wood, David Hengerer

**Affiliations:** ^1^Department of Human Development (now Department of Psychology), Cornell University, Ithaca, NY, United States; ^2^Southampton Business School, University of Southampton, Southampton, United Kingdom; ^3^Department of Psychology, Scripps College, Claremont, CA, United States; ^4^Department of Applied Cognitive Psychology, Claremont Graduate University, Claremont, CA, United States

**Keywords:** COVID-19, scam, fraud, risk taking, age

## Abstract

As the COVID-19 pandemic was unfolding, a surge in scams was registered across the globe. While COVID-19 poses higher health risks for older adults, it is unknown whether older adults are also facing higher financial risks as a result of COVID-19 scams. Here, we examined age differences in vulnerability to COVID-19 scams and individual difference measures (such as impulsivity, ad skepticism, and past experiences with fraud) that might help explain them. A lifespan sample (*M* = 48.03, *SD* = 18.56) of sixty-eight younger (18–40 years, *M* = 25.67, *SD* = 5.93), 79 middle-aged (41–64 years, *M* = 49.86, *SD* = 7.20), and 63 older adults (65–84 years, *M* = 69.87, *SD* = 4.50) recruited through Prolific completed questions and questionnaires online. In a within-subjects design, each participant responded to five COVID-19 solicitations, psychological measures, and demographic questions. Age group comparisons revealed that older adults were marginally less likely to perceive COVID-19 solicitations as genuine than middle-aged adults were. In addition, older adults perceived significantly fewer benefits than both younger and middle-aged adults did and perceived marginally higher risks than younger adults did. Hence, older adults did not exhibit greater vulnerability to COVID-19 scams. Regardless of age, intentions to respond to COVID-19 solicitations were positively predicted by higher levels of educational attainment, being married, past fraud victimization, and higher levels of positive urgency. As expected, stronger genuineness and benefit perceptions positively predicted action intentions, whereas stronger risk perceptions negatively predicted action intentions As such, COVID-19 scam susceptibility appears to be the result of a impulse control issue that is not easily inhibited, not even by past experiences of scam victimization.

## Introduction

The COVID-19 pandemic has had unparalleled impact on every facet of our lives. With 252 millions identified carriers and 5 million deaths (World Health Organization [WHO], 2021), data show that older adults are at a higher risk of experiencing COVID-19 related health complications or death (Centers for Disease Control and Prevention [CDC], 2020). Moreover, the coronavirus is not the only viral contagion of the pandemic: The emergence of COVID-19 has led to an increase in scams, with grave emotional, financial, and health consequences. We define “COVID-19 frauds or scams” as dishonest and deceitful schemes that use a COVID-19 cover story and are potentially dangerous to consumers, for instance by extracting money or personal information, selling sham products or services, or by causing bodily, technological, or other harm (e.g., through ingesting alleged healthcare products or installation of malware on electronic devices). To illustrate, such cover stories may revolve around issues caused by COVID-19 (e.g., exposure to the virus), prevention of COVID-19 (e.g., through products), or benefits associated with COVID-19 (e.g., financial relief payments).

By October 18, 2021, the United States Federal Trade Commission (FTC) reported 270,301 COVID-19 fraud cases that resulted in a total loss of over $580 million (Federal Trade Commission [FTC], 2021b). Consequently, the FTC, the Consumer Financial Protection Bureau, law enforcement agencies, and third sector organizations have all voiced warnings about scams involving COVID-19 stimulus checks, testing, treatment, vaccinations, face masks, air filters, or other COVID-19 products and services (Federal Trade Commission [FTC], 2020a,b,c). Similarly, consumers have been advised to avoid responding to COVID-19 donation scams and phishing scams from sources alleging to be the “WHO” or “CDC.”

Many warnings are specifically targeted at older adults, under the assumption that this age group is specifically vulnerable to falling prey to COVID-19 scams. After all, they are more likely to purchase or wear protective equipment (e.g., face masks), follow protective measures, seek treatment or vaccination, or donate money (Midlarsky and Hannah, 1989; Haischer et al., 2020; Barry et al., 2021). However, to our knowledge, no age-related differences in COVID-19 scam susceptibility have been established in experimental studies. In fact, the connection between age and fraud turns out to be far more complex than a simple linear relationship. One the one hand, some studies find that older adults are increasingly or even disproportionately being targeted by fraudsters (Huang and Lawitz, 2016; Burnes et al., 2017), and that victimization rates are higher among older adults, especially those who are targeted directly (AARP, 2003; James et al., 2014). Other studies, in contrast, have reported the opposite trends, finding that older adults are, in fact, less likely to fall victim to scams compared to middle-aged adults (Anderson, 2013; Flatley, 2016; Mueller et al., 2020). Divergence in findings could stem from the various types of scams or frauds used and from the fact that many consumers fail to report incidents of fraud victimization (Shao et al., 2019). In addition, age differences seem to relate to the outcome being measured. To illustrate, a report by the Federal Trade Commission [FTC] (2021a) reveals that the highest number of COVID-19 fraud complaints is being logged by adults between the ages of 30 and 39 (20%), followed by adults 40–49 years (19%) and adults 50–59 years (18%). In contrast, the median amount of money lost is highest among adults over 80 ($1,000), with younger age groups losing between $244 and $590 per person (for similar results concerning romance scams, see Fletcher, 2019). Thus, it is often difficult to gauge and verify the true rate and financial impact of fraud victimization across the lifespan.

### Theoretical Framework

From a theoretical standpoint, age-related increments in the susceptibility to COVID-19 solicitations are plausible considering that that older adults shift their focus from pursuing long-term, knowledge-focused goals to pursuing short-term, affect-focused goals (Reed and Carstensen, 2012). According to Socioemotional Selectivity Theory (Carstensen et al., 1999; Mather and Carstensen, 2005; Reed and Carstensen, 2012), these goals center around establishing and maintaining positive affect, leading older adults to exhibit a so-called “positivity effect” in their cognitive processing: Unlike younger adults, who are often more affected by negative stimuli (thus exhibiting a “negativity bias,” Kisley et al., 2007), older adults selectively focus on and remember positively valenced information better than they do negative information. Correspondingly, older adults are theorized to place greater weight on benefits than risks (Mikels et al., 2015). This, combined with a reduced ability to learn to avoid risks, may lead older adults to engage in suboptimal risk taking (e.g., Denburg et al., 2001; Mata et al., 2011; Mikels et al., 2013). As a result, older adults might pay undue attention to the benefits associated with COVID-19 solicitations while simultaneously disregarding their potential risks or costs, making older adults more likely to fall for these scams than their younger peers are.

### The Present Study

Data concerning vulnerability to fraud, particularly to COVID-19 related scams, are just starting to emerge. The present study was designed to evaluate whether age differences exist in susceptibility to COVID-19 scams, and possible mechanisms that might help explain these differences. Younger, middle-aged, and older adults were asked to indicate their intentions to respond to or interact with five solicitations based on real-life COVID-19 products or scams that may result in financial loss or information/identity theft (or even bodily harm, in the case of products that are supposed to protect against COVID-19). In addition, participants evaluated each solicitation’s perceived genuineness, benefits, and risks.

Grounded in past research, we hypothesized that a higher intention to act on COVID-19 solicitations would be predicted by higher genuineness ratings, higher benefit ratings, and lower risk ratings associated with each of the solicitations (e.g., Hanoch et al., 2006; Schoepfer and Piquero, 2009; Wood et al., 2018). Following from Socioemotional Selectivity Theory, we hypothesized that older adults would perceive higher benefits and lower risks, thus reporting increased intentions to respond to COVID-19 solicitations.

Finally, we examined the role played by individual difference measures that have been shown to be associated with susceptibility to fraud, age, or both: Previous experience with fraud (Titus and Gover, 2001; Schoepfer and Piquero, 2009), receptivity to non-sensical information (“bullshit” information, Pennycook and Rand, 2019), skepticism toward ads (Obermiller et al., 2005; MacAlvanah et al., 2015; Anderson, 2016), stress levels (Anderson, 2013), and impulsivity (AARP, 2003; Anderson, 2016; Jones et al., 2019). Specifically, we evaluated whether covariates that differ between age groups may explain the proposed age differences in scam susceptibility.

## Materials and Methods

Participants responded to a 15-min online survey that paid 2.47 USD and was implemented *via* the Qualtrics.com survey platform. IRB approval for this project was granted prior to data collection.

### Participants

United States participants were recruited through Prolific in June 2020, 3 months after the WHO identified the COVID-19 crisis as a pandemic. Prolific is an online panel provider with access to ca. vetted 40,000 participants (Palan and Schitter, 2018). Data collected through Prolific have been found to be comparable to the data collected in laboratory-based settings, with the exception that participants recruited through Prolific are more diverse as well as slightly older (Porter et al., 2018).

Among the potential participants approached, 5 dropped out after and 20 withdrew before beginning the study. Of the *N* = 231 participants we recruited, 21 were manually excluded due missing data for half or more of the survey, providing no demographic information, and/or failing attention checks; one participant was excluded because they did not provide consent prior to taking the study and should have been screened out automatically but was not. The final sample consisted of *N* = 210 participants (18–84, *M* = 48.03, *SD* = 18.56 years; see Supplementary 2A for a power analysis); three age bins were used to guarantee similar numbers of younger, middle-aged and older adults. In total, we recruited 68 younger (18–40 years, *M* = 25.67, *SD* = 5.93), 79 middle-aged (41–64 years, *M* = 49.86, *SD* = 7.20), and 63 older adults (65–84 years, *M* = 69.87, *SD* = 4.50).

### Materials

Using a within-subjects design, each participant reviewed five COVID-19 related messages and ads based on real-life scams (see see Supplementary 1A for items concerning each solicitation; the solicitation materials themselves can be obtained from the corresponding author): An email ostensibly issued by the WHO, a text message warning about alleged exposure to COVID-19, a colloidal silver (disinfectant) ad for an “all-purpose remedy” and a vaccine announcement claiming a new vaccine could cure COVID-19 in hours. Finally, to account for the possibility that some might generally distrust COVID-19 related offers, even when they are genuine, participants reviewed a legitimate face mask ad. Although some solicitations entailed potentially interactive elements (such as a button or link), participants simply reviewed pictures of each message or ad without interacting with them.

#### Demographics

Participants reported their age, gender, education, income, marital status, race/ethnicity, employment status, and political worldview. All demographic measures are provided in Supplementary 1B.

#### Individual Difference Measures

All individual difference measures were based on existing scales.

##### History of Financial Fraud

Participants self-reported whether they had ever fallen victim to financial fraud (Nolte et al., 2021).

##### Stress Symptoms and Frequency

To assess their stress level, participants were asked to respond to the K6+ Kessler Screening Scale for Psychological Distress (original Cronbach’s α = 0.89 to 0.92, Kessler et al., 2002). Stress symptoms were measured by asking how often participant had experienced six stress symptoms in the month preceding the survey, resulting in a score between 6 and 30 (Cronbach’s α = 0.92 in present sample). Stress frequency was measured by asking whether symptoms had occurred more or less often than usual (assessed on a 7-point Likert scale).^1^

##### Bullshit Receptivity

Using a 5-point Likert scale, participants evaluated the perceived profoundness of ten non-sense sentences taken from the so-called Bullshit Receptivity Scale (original Cronbach’s α = 0.82 to 0.91, Pennycook et al., 2015; Cronbach’s α = 0.94 in present sample).

##### Ad Skepticism

The degree to which participants distrust advertisements was quantified *via* an 8-item Ad Skepticism Scale (Anderson, 2016; adapted from Obermiller and Spangenberg, 1998, with original Cronbach’s α = 0.85–0.86). Responses were recorded on a 7-point Likert scale (Cronbach’s α = 0.94 in present sample).

##### Impulsivity

Participants completed the 20-item short version of the UPPS-P Impulsive Behavior Scale (original Cronbach’s α = 0.74–0.88 across subscales, Cyders et al., 2014). This scale assesses participants’ lack of premeditation, lack of perseverance, sensation seeking, positive urgency, and negative urgency using four items each (Cronbach’s α = 0.67–0.83 across subscales in present sample). Responses were scored on a 4-point Likert scale.

#### Solicitation Measures

Solicitation measures were partially adapted from Wood et al. (2018) and Nolte et al. (2021) and otherwise generated for the purpose of the present study. Cronbach’s αs based on the present sample are reported under “Data Reduction Strategy” in the section “Results.”

##### Action Intentions

Willingness to act on each solicitation was assessed by asking about participants’ likelihood of purchasing a product or clicking a link, and the likelihood of recommending a solicitation to a friend (7-point Likert scales).

##### Perceived Genuineness

Participants rated the statements “I think the [solicitation] is legitimate” and “I think the [solicitation] is deceptive” (R) on 7-point Likert scales.

##### Risk and Benefit Ratings

The risks and benefits associated with each solicitation were assessed on 7-point Likert scales.

### Procedure

After providing informed consent, participants completed questions about the five solicitations, the order of which was randomized and counterbalanced across participants. In a next step, participants responded to all individual difference measures. With the exception of the Kessler Stress Scale, which was presented first, the order of these scales was randomized and counterbalanced as well. At the end of the survey, participants provided demographic information and were debriefed about the aim of the study.

### Analyses

All analyses were conducted in RStudio Version 1.3.1093. Age group differences between younger, middle-aged, and older adults were assessed using non-parametric, rank-based between-subject ANOVAs and ANCOVAs (Results were comparable when conducting Kruskal-Wallis tests instead). Bonferroni-corrected *post hoc* tests comparing two groups at a time were conducted using independent-sample Wilcoxon tests. Regression results were based on generalized linear models, and *Pseudo-R^2^* for each regression model was calculated using the Nagelkerke formula in R’s “rcompanion” package (Mangiafico, 2020). Note that unlike *R*^2^, which indexes explained variance, *Pseudo-R^2^* may be better understood as an index of model fit. Because we tested a high number of predictors, we explicitly differentiate between findings that are significant at the conventional alpha value (0.05) and those that remain significant at a stricter alpha value (0.001).

## Results

### Data Reduction Strategy

In a first step, we compared responses between the face mask solicitation, which served as a control scenario, and the four fraudulent scenarios. To this end, we averaged participants’ action intentions (Cronbach’s α = 0.66) and ratings of perceived genuineness (Cronbach’s α = 0.52), risks (Cronbach’s α = 0.52), and benefits (Cronbach’s α = 0.73) across the four scams. Compared to the face mask solicitation, participants were less willing to respond to scams (*M*_*mask*_ = 2.44, *SD*_mask_ = 1.80, *M*_scams_ = 1.73, *SD_*scams*_* = 0.90, *V* = 2,404, *p* < 0.001) and less likely to perceive them as genuine (*M*_*mask*_ = 4.17, *SD*_mask_ = 1.54, *M*_scams_ = 2.51, *SD_*scams*_* = 0.98, *V* = 1,384.50, *p* < 0.001). In addition, the four scams were perceived to be more risky (*M*_*mask*_ = 3.71, *SD*_mask_ = 1.81, *M*_scams_ = 5.57, *SD_*scams*_* = 1.00, *V* = 16,450, *p* < 0.001) and less beneficial (*M*_*mask*_ = 3.91, *SD*_mask_ = 1.62, *M*_scams_ = 2.19, *SD_*scams*_* = 1.09, *V* = 1,227.50, *p* < 0.001). Thus, participants correctly differentiated between genuine and fraudulent solicitations.

To determine whether to include or exclude the face mask scenario in our analyses, we next examined inter-correlations for action intention, genuineness, benefit, and risk ratings across all five scenarios (see Supplementary 2B, Supplementary Table 1). Although the face mask scenario served as a control scenario, responses to this scenario showed considerable correlations with responses to the four scams (most *ps*<0.05 to <0.001). Furthermore, scale reliability for the four variables of interest (i.e., action intentions, perceived genuineness, perceived risks, perceived benefits) remained about the same or improved when including rather than excluding responses concerning the face mask solicitation. As a result, subsequent analyses were based on averages derived from all five solicitations than rather just the four fraudulent scenarios (Cronbach’s α = 0.75 action intentions, α = 0.52 perceived genuineness, α = 0.62 perceived risks, α = 0.71 perceived benefits). Supplementary Analyses for each individual solicitation are provided in Supplementary 2B, Supplementary Table 2. Supplementary Analyses based on an average of the four scams (rather than all five solicitations) are provided in Supplementary 2C, Supplementary Tables 3–6.

### Age Group Comparisons

Descriptive statistics and age group comparisons for all demographic, individual difference, and solicitation measures are provided in Table 1.

**TABLE 1:** Age group comparisons for demographic variables, individual difference measures, and solicitation measures.

Variable	Total	Younger	Middle-aged	Older	Age group comparison
				
	*M (SD)/%*	*M (SD)/%*	*M (SD)/%*	*M (SD)/%*	
***Demographic variables***					
Age	48.03 (18.56)	25.67 (5.93)^c^	49.86 (7.20)^b^	69.87 (4.50)^a^	*F*(2,207) = 809.20***, η*_*p*_^2^* = 0.89
% Female	114 (54%)	30 (44%)	48 (61%)	36 (57%)	*X*^2^(2, *N* = 208) = 3.60, *V* = 0.09
% Not Non-Hispanic White	52 (25%)	29 (43%)^a^	19 (24%)^b^	4 (6%)^c^	*X*^2^(2, *N* = 210) = 23.16***, *V* = 0.23
Education	3.50 (1.24)	3.09 (1.10)^b^	3.58 (1.17)^a^	3.84 (1.36)^a^	*F*(2,207) = 6.06**, η*_*p*_^2^* = 0.06
Income	2.68 (1.30)	2.43 (1.47)^b^	2.95 (1.26)^a^	2.60 (1.09)^ab^	*F*(2,207) = 3.99*, η*_*p*_^2^* = 0.04
% No full-time employment	129 (61%)	38 (56%)^b^	39 (49%)^b^	52 (83%)^a^	*X*^2^(2, *N* = 210) = 17.58***, *V* = 0.20
% Not married	115 (55%)	55 (81%)^a^	36 (46%)^b^	24 (38%)^b^	*X*^2^(2, *N* = 210) = 28.49***, *V* = 0.26
Political worldview	3.21 (1.76)	3.23 (1.81)	3.35 (1.64)	3.00 (1.85)	*F*(2,207) = 1.24, η*_*p*_^2^* = 0.01
***Individual difference measures***					
% Financial fraud victim	46 (22%)	12 (18%)	24 (30%)	10 (16%)	*X*^2^(2, *N* = 209) = 5.23, *V* = 0.11
Bullshit receptivity	2.13 (1.00)	2.38 (0.94)^a^	2.16 (1.06)^ab^	1.81 (0.91)^b^	*F*(2,207) = 6.11**, η*_*p*_^2^* = 0.06
Ad skepticism	4.96 (1.28)	5.09 (1.14)	4.77 (1.44)	5.04 (1.19)	*F*(2,207) = 0.70, η*_*p*_^2^* = 0.01
Stress symptoms	12.48 (5.88)	14.50 (5.31)^a^	13.19 (6.23)^a^	9.44 (4.77)^b^	*F*(2,207) = 20.16***, η*_*p*_^2^* = 0.16
Stress frequency	4.53 (1.43)	4.70 (1.58)^ab^	4.72 (1.47)^a†^	4.20 (1.22)^b†^	*F*(2,207) = 5.18**, η*_*p*_^2^* = 0.05
Lack of premeditation	1.67 (0.48)	1.75 (0.57)	1.64 (0.46)	1.62 (0.39)	*F*(2,207) = 0.87, η*_*p*_^2^* = 0.01
Lack of perseverance	1.76 (0.51)	1.79 (0.49)	1.79 (0.55)	1.67 (0.48)	*F*(2,207) = 0.92 η*_*p*_^2^* = 0.01
Sensation seeking	2.25 (0.69)	2.43 (0.69)^a^	2.24 (0.64)^ab^	2.08 (0.73)^b^	*F*(2,207) = 4.64*, η*_*p*_^2^* = 0.04
Positive urgency	1.67 (0.62)	1.78 (0.66)	1.69 (0.69)	1.52 (0.44)	*F*(2,207) = 1.93, η*_*p*_^2^* = 0.02
Negative urgency	2.09 (0.74)	2.29 (0.73)^a^	2.18 (0.75)^a^	1.78 (0.65)^b^	*F*(2,207) = 8.99***, η*_*p*_^2^* = 0.08
***Solicitation measures***					
Action intentions	1.95 (1.10)	1.90 (0.91)	2.20 (1.36)	1.69 (0.80)	*F*(2,207) = 0.71, η*_*p*_^2^* = 0.01
Perceived genuineness	2.86 (0.90)	2.79 (0.98)^ab^	3.07 (0.87)^a^^†^	2.67 (0.82)^b^^†^	*F*(2,207) = 3.12*, η*_*p*_^2^* = 0.03
Benefit rating	2.65 (1.18)	2.74 (1.02)^a^	2.91 (1.40)^a^	2.23 (0.89)^b^	*F*(2,207) = 4.75**, η*_*p*_^2^* = 0.04
Risk rating	5.13 (1.07)	5.04 (0.97)^†^	4.98 (1.21)^ab^	5.40 (0.93)^a†^	*F*(2,207) = 3.26*, η*_*p*_^2^* = 0.03

Within each row under the headings “younger,” “middle-aged,” or “older,” cells that do not share a superscript are significantly different from each other. V denotes Cramer’s V. ^†^p < 0.10, *p < 0.05, **p < 0.01, ***p < 0.001.

#### Demographics

Age groups differed with regard to their race/ethnicity (*p* < 0.001), educational attainment, income, employment status (*p* < 0.001) and marital status (*p* < 0.001; Table 1, top rows): Older adults were more likely to identify as Non-Hispanic White than both younger and middle-aged adults were, and middle-aged adults were more likely to identify as Non-Hispanic White than younger adults were. Both older and middle-aged adults reported higher educational attainment than younger adults did, and middle-aged adults reporter a higher income than younger adults did. Older adults were less likely to be in full-time employment than both younger and middle-aged adults, and younger adults were less likely to be married than both middle-aged and older adults.

#### Individual Difference Measures

With regard to individual difference measures (Table 1, middle rows), we observed differences in “bullshit” receptivity, stress symptoms (*p* < 0.001), stress frequency, sensation seeking, and negative urgency (*p* < 0.001): Younger adults proved more responsive to bullshit, reported higher stress levels, were more sensation seeking, and experienced more negative urgency than older adults did. Furthermore, middle-aged adults disclosed higher levels of negative urgency and experienced stress marginally more often than older adults did.

#### Solicitation Measures

At a conventional alpha value (0.05), age groups differed in their ratings of the solicitations’ perceived genuineness, benefits, and risks, but not in their willingness to respond to COVID-19 solicitations (Table 1, bottom rows). *Post hoc* tests revealed that older adults were marginally less likely to perceive COVID-19 solicitations as genuine than middle-aged adults were (*p* = 0.054). In addition, older adults perceived significantly fewer benefits than both younger and middle-aged adults did (*p* < 0.01) and perceived marginally higher risks than younger adults did (*p* = 0.059). None of the results reached significance at a corrected alpha value (0.001).

Next, we assessed whether demographic variables and individual difference measures could account for age-related differences in participants’ assessments of COVID-19 solicitations. To this end, we re-ran significant age group comparisons as ANCOVAs controlling for those covariates that differed between age groups at *p* < 0.05: Race/ethnicity, education, income, employment status, marital status, bullshit receptivity, stress symptoms, stress frequency, sensation seeking, and negative urgency. Age group differences in the perceived genuineness of COVID-19 solicitations were no longer significant when accounting for stress symptoms, sensation seeking, or negative urgency. Age group differences in benefit ratings were no longer significant when accounting for bullshit receptivity. Finally, age group differences in risk ratings were no longer significant when accounting for race/ethnicity, employment status, bullshit receptivity, stress symptoms, or negative urgency.

### Predicting Intentions to React in Response of a Solicitation

ANCOVA results suggested that older age is associated with various protective factors. To examine the predictive power of each assessed covariate, we regressed action intentions on all demographic, individual difference, and solicitation measures (corresponding regression models predicting perceived genuineness, benefit rating, and risk rating are provided in Supplementary 2D, Supplementary Tables 7–9). When examining each predictor separately (Table 2, middle columns), males, married participants, and those relaying a more conservative worldview were more inclined to respond to the solicitation (all *ps* < 0.05). In addition, stronger intentions were positively associated with bullshit receptivity, sensation seeking, positive urgency, negative urgency, perceived genuineness, and benefit ratings, and negatively associated with ad skepticism and risk ratings (all *ps* < 0.001).

**TABLE 2:** Regression results predicting action intentions based on demographic variables, individual difference measures, and solicitation measures.

Variable	Predictors entered Separately			Predictors entered Jointly	
	β	*p*	*Pseudo-R^2^*	β	*p*
***Demographic variables***					
Age	–0.11	0.129	0.01		
Age^2^	–0.13	0.063	0.02	0.01	0.871
% Female	–0.14	0.042	0.05	0.07	0.194
% Not Non-Hispanic White	0.11	0.114	0.01	0.04	0.406
Education	0.13	0.071	0.02	0.12	0.025
Income	0.11	0.115	0.01	0.00	0.951
% No full-time employment	–0.08	0.272	0.01	–0.06	0.241
% Not married	–0.20	0.005	0.04	–0.10	0.046
Political worldview	0.15	0.034	0.10	0.01	0.894
***Individual difference measures***					
% Financial fraud victim	0.04	0.549	0.02	0.10	0.035
Bullshit receptivity	0.47	<0.001	0.31	0.10	0.088
Ad skepticism	–0.48	<0.001	0.29	–0.10	0.107
Stress symptoms	0.13	0.067	0.06	0.10	0.146
Stress frequency	–0.12	0.171	0.71	–0.07	0.252
Lack of premeditation	0.03	0.644	0.02	–0.08	0.144
Lack of perseverance	–0.13	0.058	0.04	–0.01	0.913
Sensation seeking	0.24	<0.001	0.16	0.09	0.096
Positive urgency	0.44	<0.001	0.25	0.28	<0.001
Negative urgency	0.23	<0.001	0.06	–0.11	0.194
***Solicitation measures***					
Perceived genuineness	0.67	<0.001	0.48	0.18	0.015
Benefit rating	0.74	<0.001	0.60	0.34	<0.001
Risk rating	–0.59	<0.001	0.40	–0.21	<0.001
Intercept				0.00	0.144
*Pseudo-R^2^*				0.98	

Due to the possibility that the relationship between action intentions and age may be curvilinear (i.e., peaking in middle age), we examined both age and age^2^. Neither proved predictive of action intentions, although age^2^ approximated significance. Thus, we entered age^2^ instead of age into a joint model in which all covariates were considered simultaneously (Table 2, right columns). At a conventional alpha value (0.05), this model suggested that higher action intentions were associated with higher educational attainment, being married, having previously fallen victim to financial fraud, and perceiving the solicitations as genuine. At a stricter alpha value (0.001), action intentions were linked to higher levels of positive urgency, higher benefit ratings, and lower risk ratings. In this model, age^2^ did not approximate significance.

## Discussion

With older adults facing the most severe health risks to the virus, there have been growing worries that they might also be more vulnerable to COVID-19 scams. To address this important concern, younger, middle-aged, and older adults reported their willingness to respond to five COVID-19 solicitations obtained online.

### Age Differences in COVID-19 Scam Susceptibility

We found no consistent evidence for age differences in the willingness to respond to COVID-19 scams. At odds with Socioemotional Selectivity Theory, older adults perceived COVID-19 solicitations to offer significantly fewer benefits than both younger and middle-aged adults did. This difference was no longer significant when accounting for age differences in the susceptibility to non-sense (“bullshit”) information, which older adults appear more wary of (Erlandsson et al., 2018).

Our findings thus contribute to an emerging literature suggesting that older age might be associated with certain protective factors that might lower older adults’ vulnerability to fraud. To illustrate, a novel study finds that older adults score higher on measures of emotional understanding than those under the age of 65, and that higher levels of emotional intelligence, including higher levels of emotional understanding, shield consumers against falling for scams (Mueller et al., 2020). Other recent studies report that older adults are less amenable to persuasion and more sensitive to scam-related and financial risks than younger generations are (Rolison et al., 2014; Rolison et al., 2019; Mueller et al., 2020).

### Predictors of COVID-19 Scam Susceptibility

Our findings add to the understanding of decreased scam susceptibility in old age (see Modic and Lea, 2013; Mueller et al., 2020) but the mechanisms underlying the potentially increased scam susceptibility of younger age groups remain poorly understood. In an effort to identify potential predictors of COVID-19 scam compliance, we studied the role played by a range of individual difference measures. Various covariates predicted COVID-19 scam susceptibility when examined by themselves but only a limited number of predictors remained significant when other covariates were accounted for.

With regard to demographic variables, we found that sensu past research, educational attainment (Titus and Gover, 2001; DeLiema et al., 2017) and marital status (DeLiema et al., 2017) were associated with increased vulnerability to fraud. It has been theorized that this is because better-educated people hold “wider interests, engage in a broader range of activities, and have more consumer participation in the marketplace than other demographic groups, thereby increasing their exposure to fraudulent solicitations and transactions” (Titus and Gover, 2001, p. 133). It is possible that since marriage allows spouses to pool their finances, married consumers, too, are more likely to make certain investments or purchases that expose them to fraud attempts.

With regard to individual difference measures, we found that only two individual difference measures were associated with participants’ action intentions when also accounting for other predictors. Specifically, we found that positive urgency predicted higher action intentions across both by itself and in conjunction with other factors. Furthermore, a previous history of fraud susceptibility (Titus and Gover, 2001; Schoepfer and Piquero, 2009) emerged as a new predictor when controlling for other covariates.

Finally, with regard to solicitation-based measures, stronger action intentions were associated with stronger perceptions that a solicitation was genuine in nature, high in benefits, and low in risks (e.g., Hanoch et al., 2006; Schoepfer and Piquero, 2009; Wood et al., 2018). Thus, the observed combination of risk factors in the joint regression model suggests that scam susceptibility is the result of an impulse control issue that is not easily inhibited, not even by past experiences of scam victimization.

## Limitations

The present study is not without its limitations. First, we did not assess whether age groups differed in cognitive ability (see Shao et al., 2019), although victimization rates are higher among those with decreased capacities (James et al., 2014). However, recent research by Mueller et al. (2020) demonstrates that cognition may not play as big of a role as other predictors do in explaining why age groups differ in their likelihood of falling for scams: Decision making ability and financial literacy do not mediate the relationship between age and scam susceptibility.

The present study was also limited to the use of five COVID-19 solicitations and cannot speak to the relative persuasiveness of different types of COVID-19 scams. In addition, it is important to note that the presented solicitations were very heterogeneous in nature. As a result, it remains unclear whether certain COVID-19 products, services, or messages would be more likely to elicit age differences in scam susceptibility than the solicitations included in the present study.

Although one third of all scams are circulated *via* the internet, a considerable number of fraud attempts are made *via* telemarketing calls (9%) and print advertisements (19%) every year (Anderson, 2013). Age-related differences in scam susceptible are known to vary across scam types, with older adults being especially responsive to telemarketing, lottery, and investment scams (AARP, 2003). Therefore, results may have differed if we had conducted the present study through a different medium or face-to-face.

Finally, it is possible that our findings might not apply to the general population: Participants recruited through online panel providers, especially older ones, must be assumed to be more likely to use the internet and conduct transactions online. Thus, prior exposure to internet-based scams was highly probable, making our participants more likely to recognize online fraud attempts or to have fallen victim to such an attempt prior to the study.

## Conclusion

It is vital that warning messages are targeted and received by younger and middle-aged adults as well, not only older adults. Attempts to ward off scammers should focus on changing individuals’ genuineness, risk, and benefit perceptions, as these factors seem to be among the strongest driving forces in people’s intention to respond to scams, regardless of age.

## Data Availability Statement

The raw data, coded data, R code and variable key (for the raw data) were submitted for review alongside this manuscript. All files can be made available upon request to the first author.

## Ethics Statement

The study involving human participants was reviewed and approved by Institutional Review Board, Scripps College. Participants provided their written informed consent to participate in the study.

## Author Contributions

JN conducted the analyses and drafted the manuscript. YH, SW, and DH added to and edited the manuscript. All authors contributed to conceptualization and design of the study.

## Conflict of Interest

The authors declare that the research was conducted in the absence of any commercial or financial relationships that could be construed as a potential conflict of interest.

## Publisher’s Note

All claims expressed in this article are solely those of the authors and do not necessarily represent those of their affiliated organizations, or those of the publisher, the editors and the reviewers. Any product that may be evaluated in this article, or claim that may be made by its manufacturer, is not guaranteed or endorsed by the publisher.

## Footnotes

1 Participants also completed follow-up questions about the number of days they were unable to work or carry out normal activities, unable to carry out more than half of what they would normally be able to do, saw a doctor about their symptoms, or experienced stress symptoms due to physical causes. Due to low response rates (*ns* = 20–26), these items were dropped.
