# Correction: Susceptibility to COVID-19 scams: the roles of age, individual difference measures, and scam-related perceptions

**DOI:** 10.3389/fpsyg.2026.1840733

**Published:** 2026-04-15

**Authors:** Frontiers Production Office

**Affiliations:** Frontiers Media SA, Lausanne, Switzerland

**Keywords:** age, COVID-19, fraud, risk taking, scam

File “DataSheet 1” was erroneously published with the original version of this paper. The file has now been replaced with the correct file.

The original version of this article has been updated.

