# Supplementary material for: Susceptibility to COVID-19 Scams: The Roles of Age, Individual Difference Measures, and Scam-Related Perceptions
Source: Front Psychol. 2021 Dec 15;12:789883. doi: 10.3389/fpsyg.2021.789883 (PMC8715153; doi:10.3389/fpsyg.2021.789883)
Supplement: Supplementary file 1 [file Data_Sheet_1.DOCX]

**Susceptibility to COVID-19 Scams: The Roles of Age, Individual Difference Measures, and Scam-related Perceptions**

Julia Nolte, M.A., M.Sc.^1*^, Yaniv Hanoch, Ph.D.^2^, Stacey Wood, Ph.D.^3^, David Hengerer. M.A.^4^

^1^Human Development Department, Cornell University**

^2^Southampton Business School, University of Southampton

^3^Department of Psychology, Scripps College

^4^Department of Applied Cognitive Psychology, Claremont Graduate University

**Correspondence:***Julia Nolte

jn472@cornell.edu

** The Cornell University Department of Human Development is now the Department of Psychology.

Supplementary Material

# Supplement 1: Study Materials

Supplement 1A: Solicitation measures

Supplement 1B: Demographic questions

**Supplement 2: Supplementary Analyses**

Supplement 2A: Power analysis

Supplement 2B: Results for each individual solicitation

Supplement 2C: Results based on the four fraudulent solicitations only

Supplement 2D: Supplementary regression models for all five solicitations

# Supplement 1: Study Materials

Supplement 1A: Solicitation measures


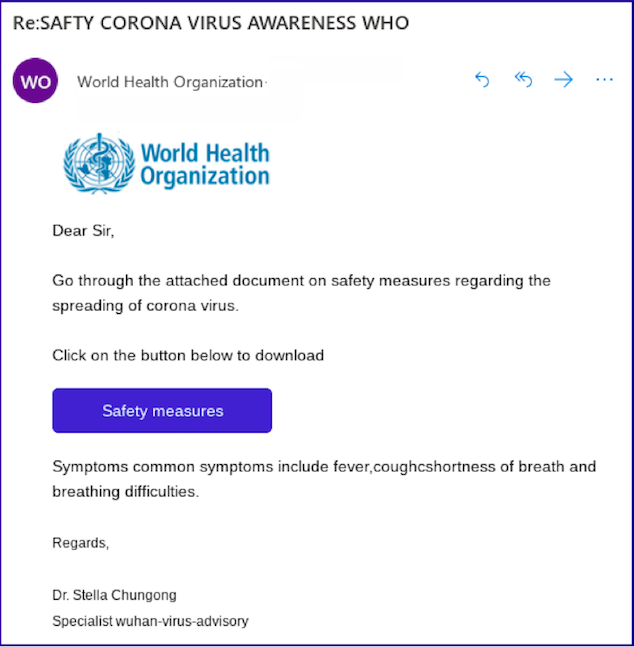


**Supplementary Figure 1.** World Health Organization (WHO) solicitation.

**Below are several brief statements that may describe your views of the email to one degree or another.  On a scale of strongly agree to strongly disagree, please indicate how much you agree or disagree with the statement.  There are no right or wrong answers to these questions, we just want to know your opinions about the email.**

|  | 7 | 6 | 5 | 4 | 3 | 2 | 1 |
| --- | --- | --- | --- | --- | --- | --- | --- |
| I think the email is legitimate | o | o | o | o | o | o | o |
| I think the email is deceptive (R) | o | o | o | o | o | o | o |

7 = Strongly Agree, 6 = Agree, 5 = Somewhat Agree, 4 = Neither Agree nor Disagree, 3 = Somewhat Disagree, 2 = Disagree, 1 = Strongly Disagree

**How likely are you to:**

|  | 7 | 6 | 5 | 4 | 3 | 2 | 1 |
| --- | --- | --- | --- | --- | --- | --- | --- |
| Click the button labeled “Safety measures”? |  |  |  |  |  |  |  |
| Forward this email on to a friend and suggest they click the button? |  |  |  |  |  |  |  |

7 = Extremely likely, 6 = Moderately likely, 5 = Slightly likely, 4 = Neither likely nor unlikely, 3 = Slightly unlikely, 2 = Moderately unlikely, 1 = Extremely unlikely

**For the following 2 questions, please rate the relative level of risk/benefit you perceive to be associated with clicking the button in the email.**

|  | 1 | 2 | 3 | 4 | 5 | 6 | 7 |
| --- | --- | --- | --- | --- | --- | --- | --- |
| Risk? |  |  |  |  |  |  |  |
| Benefit? |  |  |  |  |  |  |  |

1 = Very Low, 2 = Low, 3 = Somewhat Low, 4 = Neither Low nor High, 5 = Somewhat High, 6 = High, 7 = Very High


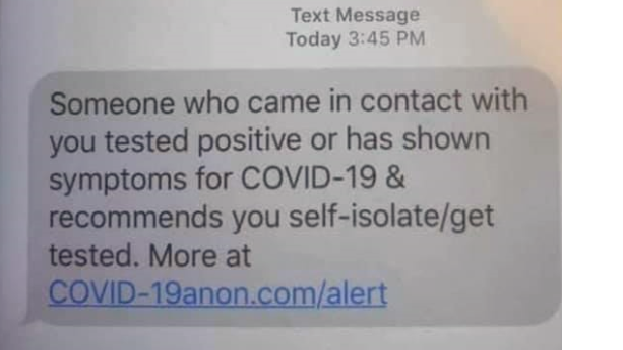


**Supplementary Figure 2.** COVID-19 exposure solicitation.

**Below are several brief statements that may describe your views of the text message to one degree or another. On a scale of strongly agree to strongly disagree, please indicate how much you agree or disagree with the statement.  There are no right or wrong answers to these questions, we just want to know your opinions about the text message.**

|  | 7 | 6 | 5 | 4 | 3 | 2 | 1 |
| --- | --- | --- | --- | --- | --- | --- | --- |
| I think the test message is legitimate | o | o | o | o | o | o | o |
| I think the text message is deceptive (R) | o | o | o | o | o | o | o |

7 = Strongly Agree, 6 = Agree, 5 = Somewhat Agree, 4 = Neither Agree nor Disagree, 3 = Somewhat Disagree, 2 = Disagree, 1 = Strongly Disagree

**How likely are you to:**

|  | 7 | 6 | 5 | 4 | 3 | 2 | 1 |
| --- | --- | --- | --- | --- | --- | --- | --- |
| Click the link in the text message? |  |  |  |  |  |  |  |
| Forward the link on to a friend? |  |  |  |  |  |  |  |

7 = Extremely likely, 6 = Moderately likely, 5 = Slightly likely, 4 = Neither likely nor unlikely, 3 = Slightly unlikely, 2 = Moderately unlikely, 1 = Extremely unlikely

**For the following 2 questions, please rate the relative level of risk/benefit you perceive to be associated with following the link in the text message.**

|  | 1 | 2 | 3 | 4 | 5 | 6 | 7 |
| --- | --- | --- | --- | --- | --- | --- | --- |
| Risk? |  |  |  |  |  |  |  |
| Benefit? |  |  |  |  |  |  |  |

1 = Very Low, 2 = Low, 3 = Somewhat Low, 4 = Neither Low nor High, 5 = Somewhat High, 6 = High, 7 = Very High


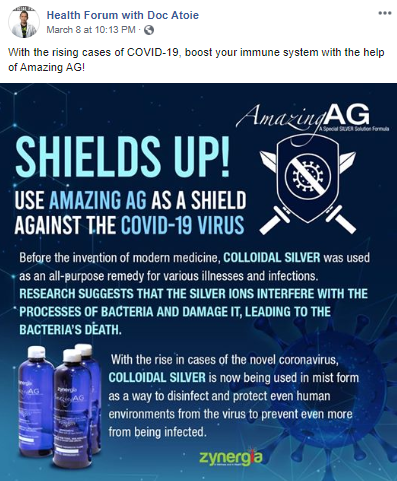


**Supplementary Figure 3.** Colloidal silver solicitation.

**Below are several brief statements that may describe your views of the advertisement to one degree or another.  On a scale of strongly agree to strongly disagree, please indicate how much you agree or disagree with the statement.  There are no right or wrong answers to these questions, we just want to know your opinions about the advertisement.**

|  | 7 | 6 | 5 | 4 | 3 | 2 | 1 |
| --- | --- | --- | --- | --- | --- | --- | --- |
| I think the ad is legitimate | o | o | o | o | o | o | o |
| I think the ad is deceptive (R) | o | o | o | o | o | o | o |

7 = Strongly Agree, 6 = Agree, 5 = Somewhat Agree, 4 = Neither Agree nor Disagree, 3 = Somewhat Disagree, 2 = Disagree, 1 = Strongly Disagree

**How likely are you to:**

|  | 7 | 6 | 5 | 4 | 3 | 2 | 1 |
| --- | --- | --- | --- | --- | --- | --- | --- |
| Purchase the product shown in the ad? |  |  |  |  |  |  |  |
| Recommend the product to a friend? |  |  |  |  |  |  |  |

7 = Extremely likely, 6 = Moderately likely, 5 = Slightly likely, 4 = Neither likely nor unlikely, 3 = Slightly unlikely, 2 = Moderately unlikely, 1 = Extremely unlikely

**For the following 2 questions, please rate the relative level of risk/benefit you perceive to be associated with the product shown in the ad**

|  | 1 | 2 | 3 | 4 | 5 | 6 | 7 |
| --- | --- | --- | --- | --- | --- | --- | --- |
| Risk? |  |  |  |  |  |  |  |
| Benefit? |  |  |  |  |  |  |  |

1 = Very Low, 2 = Low, 3 = Somewhat Low, 4 = Neither Low nor High, 5 = Somewhat High, 6 = High, 7 = Very High


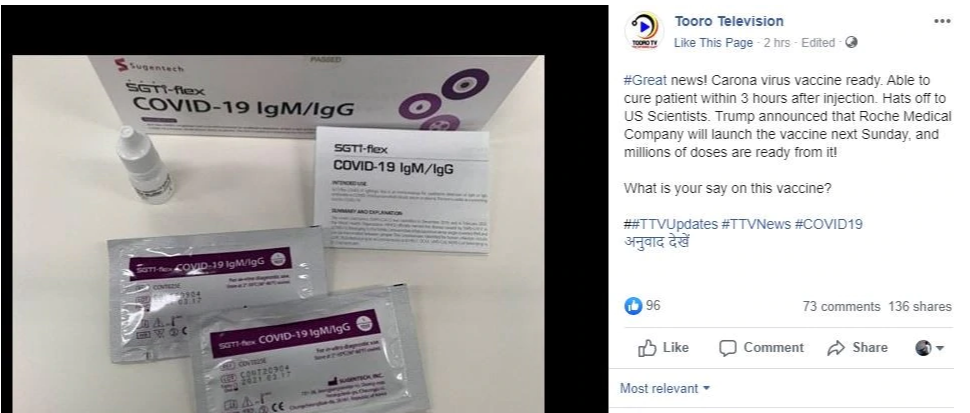


**Supplementary Figure 4.** *Vaccine solicitation.*

**Below are several brief statements that may describe your views of the social media post to one degree or another. On a scale of strongly agree to strongly disagree, please indicate how much you agree or disagree with the statement.  There are no right or wrong answers to these questions, we just want to know your opinions about the social media post.**

|  | 7 | 6 | 5 | 4 | 3 | 2 | 1 |
| --- | --- | --- | --- | --- | --- | --- | --- |
| I think the social media post is legitimate | o | o | o | o | o | o | o |
| I think the social media post is deceptive (R) | o | o | o | o | o | o | o |

7 = Strongly Agree, 6 = Agree, 5 = Somewhat Agree, 4 = Neither Agree nor Disagree, 3 = Somewhat Disagree, 2 = Disagree, 1 = Strongly Disagree

**How likely are you to:**

|  | 7 | 6 | 5 | 4 | 3 | 2 | 1 |
| --- | --- | --- | --- | --- | --- | --- | --- |
| Purchase the product shown in the social media post? |  |  |  |  |  |  |  |
| Recommend the product to a friend? |  |  |  |  |  |  |  |

7 = Extremely likely, 6 = Moderately likely, 5 = Slightly likely, 4 = Neither likely nor unlikely, 3 = Slightly unlikely, 2 = Moderately unlikely, 1 = Extremely unlikely

**For the following 2 questions, please rate the relative level of risk/benefit you perceive to be associated with the product shown in the social media post.**

|  | 1 | 2 | 3 | 4 | 5 | 6 | 7 |
| --- | --- | --- | --- | --- | --- | --- | --- |
| Risk? |  |  |  |  |  |  |  |
| Benefit? |  |  |  |  |  |  |  |

1 = Very Low, 2 = Low, 3 = Somewhat Low, 4 = Neither Low nor High, 5 = Somewhat High, 6 = High, 7 = Very High


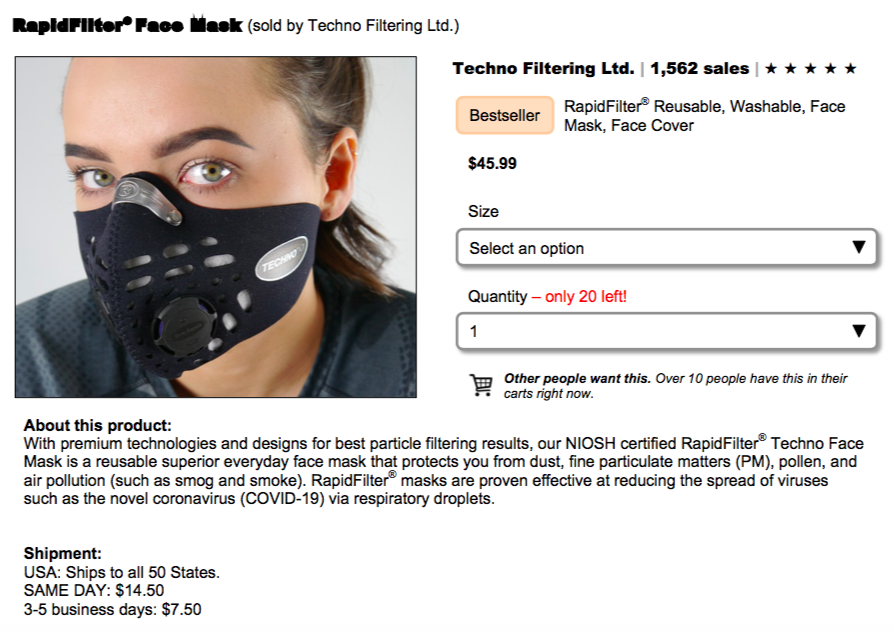


**Supplementary Figure 5.** *Face mask solicitation.*

**Below are several brief statements that may describe your views of the advertisement to one degree or another. On a scale of strongly agree to strongly disagree, please indicate how much you agree or disagree with the statement. There are no right or wrong answers to these questions, we just want to know your opinions about the advertisement.**

|  | 7 | 6 | 5 | 4 | 3 | 2 | 1 |
| --- | --- | --- | --- | --- | --- | --- | --- |
| I think the ad is legitimate | o | o | o | o | o | o | o |
| I think the ad is deceptive (R) | o | o | o | o | o | o | o |

7 = Strongly Agree, 6 = Agree, 5 = Somewhat Agree, 4 = Neither Agree nor Disagree, 3 = Somewhat Disagree, 2 = Disagree, 1 = Strongly Disagree

**How likely are you to:**

|  | 7 | 6 | 5 | 4 | 3 | 2 | 1 |
| --- | --- | --- | --- | --- | --- | --- | --- |
| Purchase this face mask? |  |  |  |  |  |  |  |
| Recommend the face mask to a friend? |  |  |  |  |  |  |  |

7 = Extremely likely, 6 = Moderately likely, 5 = Slightly likely, 4 = Neither likely nor unlikely, 3 = Slightly unlikely, 2 = Moderately unlikely, 1 = Extremely unlikely

**For the following 2 questions, please rate the relative level of risk/benefit you perceive to be associated with purchasing the mask directly from the ad.**

|  | 1 | 2 | 3 | 4 | 5 | 6 | 7 |
| --- | --- | --- | --- | --- | --- | --- | --- |
| Risk? |  |  |  |  |  |  |  |
| Benefit? |  |  |  |  |  |  |  |

1 = Very Low, 2 = Low, 3 = Somewhat Low, 4 = Neither Low nor High, 5 = Somewhat High, 6 = High, 7 = Very High

Supplement 1A: Demographic questions

**What is your age?** ______

**What is your gender?**

Male

Female

I do not identify with the options given

**What is your ethnicity? (Choose all that apply)**

White

Native Indian or Alaska Native

Asian/ Pacific Islander

Black or African American

Hispanic or Latino

Other: ______

**What is your highest level of education completed?**

Do not have high school degree or GED (1)

High school degree/ GED (2)

Associate’s degree (3)

Bachelor’s degree (4)

Master’s degree (5)

Professional degree (MD, JD, etc.) (6)

Ph.D. (7)

**What is your annual income?**

$0 - $24,999 (1)

$25,000 - $49,999 (2)

$50,000 - $74,999 (3)

$75,000 - $124,999 (4)

$125,000 - $174,999 (5)

$175,000+ (6)

**Which of the following best describes your worldview?**

Very Liberal (1)

Liberal (2)

Liberal/ Moderate (3)

Moderate (4)

Moderate/ Conservative (5)

Conservative (6)

Very Conservative (7)

Prefer not to say (NA)

**What is your employment status?**

Full-time

Part-time

Unemployed

Student

**What is your marital status?**

Single

Married

Divorced

Widowed

# Supplement 2: Supplementary Analyses

Supplement 2A: Power analysis

A post-hoc power analysis for one-way *F*-style analyses suggested that assuming *α* = .05 and three age groups, a sample of *N* = 210 was sufficiently large to detect an effect of small-to-medium size (*f* = .22, with *f* = .10 representing a small effect size and *f* =. .25 representing a medium effect size) with a power of > .80 when comparing younger, middle-aged, and older adults.

A post-hoc power analysis for linear multiple regression models suggested that assuming *α* = .05, a sample of *N* = 210 was sufficiently large to detect an effect of small-to-medium size (*f^2^* = .04, with *f^2^* = .02 representing a small effect size and *f^2^* = .15 representing a medium effect size) with a power of > .80 when regressing outcome measures on age and other predictors.

Supplement 2B: Results for individual solicitations

**Supplementary Table 1**

*Spearman’s rho (r_s_) Correlations among the Five Solicitations for Action Intentions, Perceived Genuineness, Benefit Rating, and Risk Rating*

|  | **WHO** | **COVID-19 Exposure** | **Colloidal Silver** | **Vaccine** | **Face Mask** |
| --- | --- | --- | --- | --- | --- |
| *Action intentions* |  |  |  |  |  |
| WHO | 1.00 |  |  |  |  |
| COVID-19 Exposure | .39*** | 1.00 |  |  |  |
| Colloidal Silver | .29*** | .23** | 1.00 |  |  |
| Vaccine | .44*** | .32*** | .41*** | 1.00 |  |
| Face Mask | .34*** | .33*** | .30*** | .27*** | 1.00 |
| *Perceived genuineness* |  |  |  |  |  |
| WHO | 1.00 |  |  |  |  |
| COVID-19 Exposure | .20** | 1.00 |  |  |  |
| Colloidal Silver | .28*** | .04 | 1.00 |  |  |
| Vaccine | .35*** | .27*** | .36*** | 1.00 |  |
| Face Mask | .17* | .12 | .14* | .01 | 1.00 |
| *Benefit rating* |  |  |  |  |  |
| WHO | 1.00 |  |  |  |  |
| COVID-19 Exposure | .40*** | 1.00 |  |  |  |
| Colloidal Silver | .34*** | .20** | 1.00 |  |  |
| Vaccine | .47*** | .32*** | .47*** | 1.00 |  |
| Face Mask | .20** | .29*** | .20** | .16* | 1.00 |
| *Risk rating* |  |  |  |  |  |
| WHO | 1.00 |  |  |  |  |
| COVID-19 Exposure | .32*** | 1.00 |  |  |  |
| Colloidal Silver | .30*** | .13 | 1.00 |  |  |
| Vaccine | .24*** | .19** | .40*** | 1.00 |  |
| Face Mask | .18* | .11 | .21** | .27*** | 1.00 |

*Note.* **p* < .05, ***p* < .01, ****p* < .001

**Supplementary Table 2**

# *Descriptive Statistics and Age Group Comparisons for Each of the Five COVID-19 solicitations*

|  | Genuineness | Benefit | Risk | Action Intentions |
| --- | --- | --- | --- | --- |
|  | *M (SD)* | *M (SD)* | *M (SD)* | *M (SD)* |
| *WHO* | *X^2^*(2, *N* = 207) = 4.34 | *X^2^*(2, *N* = 210) = 1.04 | *X^2^*(2, *N* = 210) =.75 | *X^2^*(2, *N* = 203) =.45 |
| Younger | 2.30 (1.76) | 2.38 (1.72) | 5.40 (1.93) | 1.73 (1.48) |
| Middle-aged | 2.65 (1.51) | 2.37 (2.04) | 5.61 (1.90) | 2.16 (1.96) |
| Older | 2.79 (1.82) | 2.21 (1.76) | 5.46 (2.09) | 1.91 (1.78) |
| *COVID-19 Exp.* | *X^2^*(2, *N* = 207) = 2.45 | *X^2^*(2, *N* =210) = 5.12 | *X^2^*(2, *N* = 210) = 1.59 | *X^2^*(2, *N* = 209) = 2.43 |
| Younger | 3.20 (1.82) | 2.96 (1.98) | 5.12 (1.70) | 2.22 (1.56) |
| Middle-aged | 3.52 (1.68) | 3.43 (2.11) | 4.87 (1.94) | 2.57 (1.98) |
| Older | 3.05 (1.68) | 2.71 (1.89) | 5.29 (1.77) | 2.15 (1.72) |
| *Colloidal Silver* | *X^2^*(2, *N* = 208) = 4.11 | *X^2^*(2, *N* = 210) = 5.25 | *X^2^*(2, *N* = 210) =.74 | *X^2^* (2, *N* = 206) = 6.01* |
| Younger | 2.28 (1.45) | 2.10 (1.36) | 5.28 (1.57) | 1.38 (.96)^ab^ |
| Middle-aged | 2.69 (1.48) | 2.44 (1.89) | 5.05 (1.86) | 1.73 (1.41)^a†^ |
| Older | 2.32 (1.50) | 1.79 (1.38) | 5.33 (1.68) | 1.30 (.89)^b†^ |
| *Vaccine* | *X^2^*(2, *N* = 201) = 9.36*** | *X^2^*(2, *N* = 210) = 11.99** | *X^2^*(2, *N* = 210) = 7.30* | *X^2^* (2, *N* = 205) = 5.70 |
| Younger | 1.86 (1.36)^a^ | 1.96 (1.57)^a^ | 6.04 (1.40)^ab^ | 1.42 (.99) |
| Middle-aged | 1.96 (1.33)^a^ | 2.27 (1.95)^a^ | 5.79 (1.55)^b^ | 1.62 (1.32) |
| Older | 1.37 (.88)^b^ | 1.43 (1.24)^b^ | 6.44 (.98)^a^ | 1.14 (.58) |
| *Face Mask* | *X^2^*(2, *N* = 209) = 7.80* | *X^2^*(2, *N* = 209) = 23.74*** | *X^2^*(2, *N* = 210) = 13.84*** | *X^2^* (2, *N* = 207) = 11.87*** |
| Younger | 4.16 (1.66)^ab^ | 4.28 (1.56)^a^ | 3.34 (1.63)^b^ | 2.60 (1.83)^a^ |
| Middle-aged | 4.53 (1.24)^a^ | 4.26 (1.59)^a^ | 3.47 (1.86)^b^ | 2.89 (2.00)^a^ |
| Older | 3.74 (1.67)^b^ | 3.08 (1.43)^b^ | 4.43 (1.75)^a^ | 1.72 (1.22)^b^ |

*Note. X^2^* tests represent Kruskal-Wallis *X^2^* tests. Within each row and column combination (e.g., genuineness ratings for the vaccine solicitation), cells that do not share a superscript are significantly different from each other; pairwise comparisons rely on Bonferroni-correction. ^†^ *p* < .10, * *p* < .05, ** *p* < .01, *** *p* < .001

Supplement 2C: Results based on the four fraudulent solicitations only

Age groups did not differ in their willingness to respond to the four fraudulent COVID-19 solicitations, *F*(2, 207) = .56, p = .570, *η_p_^2^ =* .01. In addition, we observed no age group differences with regard to perceived genuineness (*F*(2, 207) = 1.89, p = .154, *η_p_^2^ =* .02), perceived benefits (*F*(2, 207) = 1.07, p = .345, *η_p_^2^ =* .01), or perceived risks (*F*(2, 207) = .98, p = .377, *η_p_^2^ =* .01) of COVID-19 fraud attempts.

**Supplementary Table 3**

*Regression Results Predicting Action Intentions for the Four Fraudulent Solicitations Based on Demographic Variables, Individual Difference Measures, and Solicitation Measures*

| **Variable** |  | **Predictors Entered**  **Separately** | | | **Predictors Entered Jointly** | |
| --- | --- | --- | --- | --- | --- | --- |
|  |  | ***β*** | ***p*** | ***Pseudo-R^2^*** | ***β*** | ***p*** |
| *Demographic variables* | |  |  |  |  |  |
| Age | | -.02 | .805 | .00 | .06 | .481 |
| Age^2^ | | -.02 | .766 | .00 | - | - |
| % Female | | .01 | .909 | .01 | .08 | .246 |
| % Not Non-Hispanic White | | .12 | .102 | .02 | .08 | .240 |
| Education |  | .11 | .123 | .11 | .114 |  |
| Income | | .03 | .705 | .00 | .00 | .987 |
| % No full-time employment | | -.04 | .560 | .00 | -.03 | .619 |
| % Not married | | -.13 | .085 | .02 | -.08 | .286 |
| Political worldview | | .11 | .127 | .10 | -.01 | .909 |
| *Individual difference measures* | |  |  |  |  |  |
| % Financial fraud victim | | .07 | .363 | .01 | .06 | .349 |
| Bullshit receptivity | | .35 | < .001 | .22 | .12 | .106 |
| Ad skepticism | | -.32 | < .001 | .16 | -.03 | .715 |
| Stress symptoms | | .06 | .415 | .04 | -.03 | .757 |
| Stress frequency | | -.20 | .025 | .74 | -.02 | .805 |
| Lack of premeditation | | .04 | .560 | .02 | -.07 | .371 |
| Lack of perseverance | | -.11 | .139 | .02 | -.08 | .254 |
| Sensation seeking | | .09 | .221 | .05 | .06 | .339 |
| Positive urgency | | .22 | .002 | .09 | .22 | .026 |
| Negative urgency | | .08 | .255 | .01 | -.06 | .570 |
| *Solicitation measures* | |  |  |  |  |  |
| Perceived genuineness | | .69 | < .001 | .55 | .25 | .020 |
| Benefit rating | | .73 | < .001 | .66 | .31 | .003 |
| Risk rating | | -.57 | < .001 | .50 | -.27 | .002 |
| Intercept | |  |  |  | .00 | .064 |
| *Pseudo-R^2^* | |  | | | .98 | |

**Supplementary Table 4**

*Regression Results Predicting Perceived Genuineness for the Four Fraudulent Solicitations Based on Demographic Variables, Individual Difference Measures, and Solicitation Measures*

| **Variable** |  | **Predictors Entered**  **Separately** | | | **Predictors Entered Jointly** | |
| --- | --- | --- | --- | --- | --- | --- |
|  |  | ***β*** | ***p*** | ***Pseudo-R^2^*** | ***β*** | ***p*** |
| *Demographic variables* | |  |  |  |  |  |
| Age | | -.01 | .869 | .00 | .05 | .569 |
| Age^2^ | | -.02 | .730 | .00 |  |  |
| % Female | | -.03 | .694 | .03 | -.03 | .683 |
| % Not Non-Hispanic White | | .08 | .249 | .01 | .00 | .973 |
| Education |  | .06 | .385 | .00 | .00 | .975 |
| Income | | .02 | .780 | .00 | -.03 | .703 |
| % No full-time employment | | .08 | .250 | .01 | .06 | .330 |
| % Not married | | -.16 | .025 | .03 | -.02 | .733 |
| Political worldview | | .19 | .006 | .12 | .07 | .307 |
| *Individual difference measures* | |  |  |  |  |  |
| % Financial fraud victim | | .05 | .453 | .01 | -,03 | .685 |
| Bullshit receptivity | | .31 | < .001 | .18 | ,03 | .649 |
| Ad skepticism | | -.34 | < .001 | .18 | -.06 | .381 |
| Stress symptoms | | .03 | .686 | .04 | .11 | .285 |
| Stress frequency | | -.19 | .019 | .68 | -.05 | .542 |
| Lack of premeditation | | .04 | .608 | .02 | .13 | .066 |
| Lack of perseverance | | -.10 | .154 | .03 | -.08 | .280 |
| Sensation seeking | | .00 | .972 | .03 | -.09 | .155 |
| Positive urgency | | .27 | < .001 | .12 | .00 | .963 |
| Negative urgency | | .15 | .027 | .03 | .06 | .532 |
| *Solicitation measures* | |  |  |  |  |  |
| Action intentions | | .69 | < .001 | .67 | .24 | .020 |
| Benefit rating | | .77 | < .001 | .71 | .45 | < .001 |
| Risk rating | | -.57 | < .001 | .44 | -.15 | .083 |
| Intercept | |  |  |  | .00 | .064 |
| *Pseudo-R^2^* | |  | | | .98 | |

**Supplementary Table 5**

*Regression Results Predicting Benefit Rating for the Four Fraudulent Solicitations Based on Demographic Variables, Individual Difference Measures, and Solicitation Measures*

| **Variable** |  | **Predictors Entered**  **Separately** | | | **Predictors Entered Jointly** | |
| --- | --- | --- | --- | --- | --- | --- |
|  |  | ***β*** | ***p*** | ***Pseudo-R^2^*** | ***β*** | ***p*** |
| *Demographic variables* | |  |  |  |  |  |
| Age | | -.12 | .086 | .02 | -.12 | .187 |
| Age^2^ | | -.13 | .081 | .02 |  |  |
| % Female | | -.05 | .512 | .03 | -.08 | .310 |
| % Not Non-Hispanic White | | .12 | .095 | .02 | -.02 | .737 |
| Education |  | .05 | .478 | .00 | -.02 | .838 |
| Income | | -.02 | .812 | .00 | .05 | .453 |
| % No full-time employment | | .02 | .834 | .00 | .03 | .660 |
| % Not married | | -.09 | .215 | .01 | .05 | .484 |
| Political worldview | | .17 | .020 | .15 | -.09 | .202 |
| *Individual difference measures* | |  |  |  |  |  |
| % Financial fraud victim | | .06 | .429 | .02 | -.02 | .706 |
| Bullshit receptivity | | .37 | < .001 | .19 | .02 | .805 |
| Ad skepticism | | -.32 | < .001 | .18 | .00 | .949 |
| Stress symptoms | | -.01 | .854 | .04 | -.18 | .086 |
| Stress frequency | | -.17 | .048 | .73 | .03 | .727 |
| Lack of premeditation | | .01 | .847 | .00 | -.06 | .458 |
| Lack of perseverance | | -.10 | .170 | .02 | .04 | .581 |
| Sensation seeking | | .05 | .448 | .06 | .03 | .614 |
| Positive urgency | | .22 | .002 | .12 | -.07 | .473 |
| Negative urgency | | .15 | .032 | .02 | .06 | .584 |
| *Solicitation measures* | |  |  |  |  |  |
| Action intentions | | .73 | < .001 | .70 | .32 | .003 |
| Perceived genuineness | | .77 | < .001 | .66 | .50 | < .001 |
| Risk rating | | -.63 | < .001 | .50 | -.11 | .210 |
| Intercept | |  |  |  | .00 | .152 |
| *Pseudo-R^2^* | |  | | | .98 | |

**Supplementary Table 6**

*Regression Results Predicting Risk Rating for the Four Fraudulent Solicitations Based on Demographic Variables, Individual Difference Measures, and Solicitation Measures*

| **Variable** |  | **Predictors Entered**  **Separately** | | | **Predictors Entered Jointly** | |
| --- | --- | --- | --- | --- | --- | --- |
|  |  | ***β*** | ***p*** | ***Pseudo-R^2^*** | ***β*** | ***p*** |
| *Demographic variables* | |  |  |  |  |  |
| Age | | .07 | .314 | .01 | .06 | .554 |
| Age^2^ | | .06 | .368 | .00 |  |  |
| % Female | | -.12 | .080 | .04 | -.13 | .142 |
| % Not Non-Hispanic White | | -.12 | .080 | .02 | .02 | .820 |
| Education |  | -.03 | .624 | .00 | -.01 | .945 |
| Income | | .08 | .273 | .01 | .10 | .244 |
| % No full-time employment | | -.02 | .809 | .00 | -.01 | .884 |
| % Not married | | -.10 | .147 | .01 | -.09 | .313 |
| Political worldview | | -.12 | .108 | .09 | -.11 | .229 |
| *Individual difference measures* | |  |  |  |  |  |
| % Financial fraud victim | | -.08 | .237 | .02 | -.01 | .873 |
| Bullshit receptivity | | -.16 | .026 | .09 | .14 | .122 |
| Ad skepticism | | .17 | .015 | .10 | .03 | .691 |
| Stress symptoms | | .03 | .673 | .04 | -.10 | .400 |
| Stress frequency | | .19 | .021 | .73 | .07 | .483 |
| Lack of premeditation | | -.11 | .121 | .03 | .01 | .910 |
| Lack of perseverance | | -.02 | .817 | .02 | -.16 | .060 |
| Sensation seeking | | .08 | .260 | .04 | .05 | .525 |
| Positive urgency | | -.09 | .221 | .06 | .06 | .611 |
| Negative urgency | | -.06 | .417 | .00 | .07 | .591 |
| *Solicitation measures* | |  |  |  |  |  |
| Action intentions | | -.57 | < .001 | .54 | -.39 | .002 |
| Perceived genuineness | | -.57 | < .001 | .36 | -.23 | .082 |
| Benefit rating | | -.53 | < .001 | .38 | -.16 | .210 |
| Intercept | |  |  |  | .00 | < .001 |
| *Pseudo-R^2^* | |  | | | .96 | |

Supplement 2D: Supplementary regression models for all five solicitations

**Supplementary Table 7**

*Regression Results Predicting Perceived Genuineness Based on Demographic Variables, Individual Difference Measures, and Solicitation Measures*

| **Variable** |  | **Predictors Entered**  **Separately** | | | **Predictors Entered Jointly** | |
| --- | --- | --- | --- | --- | --- | --- |
|  |  | ***β*** | ***p*** | ***Pseudo-R^2^*** | ***β*** | ***p*** |
| *Demographic variables* | |  |  |  |  |  |
| Age | | -.06 | .428 | .00 | .10 | .280 |
| Age^2^ | | -.08 | .269 | .01 |  |  |
| % Female | | -.03 | .619 | .03 | -.01 | .935 |
| % Not Non-Hispanic White | | .05 | .507 | .00 | .01 | .883 |
| Education |  | .03 | .656 | .00 | -.13 | .075 |
| Income | | .01 | .916 | .00 | -.08 | .274 |
| % No full-time employment | | .02 | .764 | .00 | .05 | .397 |
| % Not married | | -.14 | .037 | .02 | .01 | .843 |
| Political worldview | | .19 | .008 | .13 | .00 | .967 |
| *Individual difference measures* | |  |  |  |  |  |
| % Financial fraud victim | | -.10 | .665 | .00 | -.07 | .273 |
| Bullshit receptivity | | .31 | < .001 | .18 | -.05 | .511 |
| Ad skepticism | | -.37 | < .001 | .20 | .04 | .598 |
| Stress symptoms | | .02 | .798 | .05 | .02 | .833 |
| Stress frequency | | -.15 | .073 | .65 | -.09 | .237 |
| Lack of premeditation | | .00 | .955 | .01 | .17 | .021 |
| Lack of perseverance | | -.14 | .051 | .04 | -.11 | .126 |
| Sensation seeking | | .03 | .632 | .08 | -.10 | .173 |
| Positive urgency | | .23 | < .001 | .10 | -.22 | .040 |
| Negative urgency | | .15 | .031 | .02 | .23 | .033 |
| *Solicitation measures* | |  |  |  |  |  |
| Action intentions | | .67 | < .001 | .53 | .32 | .016 |
| Benefit rating | | .71 | < .001 | .56 | .48 | < .001 |
| Risk rating | | -.58 | < .001 | .39 | -.14 | .085 |
| Intercept | |  |  |  | .00 | .004 |
| *Pseudo-R^2^* | |  | | | .96 | |

**Supplementary Table 8**

*Regression Results Predicting Benefit Rating Based on Demographic Variables, Individual Difference Measures, and Solicitation Measures*

| **Variable** |  | **Predictors Entered**  **Separately** | | | **Predictors Entered Jointly** | |
| --- | --- | --- | --- | --- | --- | --- |
|  |  | ***β*** | ***p*** | ***Pseudo-R^2^*** | ***β*** | ***p*** |
| *Demographic variables* | |  |  |  |  |  |
| Age | | -.18 | .009 | .03 | -.16 | .028 |
| Age^2^ | | -.21 | .003 | .04 |  |  |
| % Female | | -.10 | .147 | .04 | -.02 | .734 |
| % Not Non-Hispanic White | | .09 | .180 | .01 | -.02 | .764 |
| Education |  | .02 | .802 | .00 | .06 | .324 |
| Income | | .07 | .288 | .01 | .08 | .167 |
| % No full-time employment | | -.06 | .396 | .00 | .04 | .443 |
| % Not married | | -.09 | .200 | .01 | .03 | .609 |
| Political worldview | | .13 | .069 | .12 | -.06 | .268 |
| *Individual difference measures* | |  |  |  |  |  |
| % Financial fraud victim | | .06 | .381 | .00 | -.01 | .903 |
| Bullshit receptivity | | .47 | < .001 | .33 | .06 | .376 |
| Ad skepticism | | -.48 | < .001 | .34 | -.11 | .108 |
| Stress symptoms | | .06 | .417 | .04 | -.16 | .048 |
| Stress frequency | | -.09 | .269 | .71 | .10 | .152 |
| Lack of premeditation | | -.02 | .775 | .01 | -.04 | .500 |
| Lack of perseverance | | -.18 | .011 | .06 | .04 | .522 |
| Sensation seeking | | .19 | .008 | .11 | .02 | .738 |
| Positive urgency | | .32 | < .001 | .15 | .09 | .351 |
| Negative urgency | | .19 | .005 | .04 | -.06 | .516 |
| *Solicitation measures* | |  |  |  |  |  |
| Action intentions | | .74 | < .001 | .63 | .45 | < .001 |
| Perceived genuineness | | .71 | < .001 | .53 | .35 | < .001 |
| Risk rating | | -.58 | < .001 | .40 | -.03 | .679 |
| Intercept | |  |  |  | .00 | .234 |
| *Pseudo-R^2^* | |  | | | .98 | |

**Supplementary Table 9**

*Regression Results Predicting Risk Rating Based on Demographic Variables, Individual Difference Measures, and Solicitation Measures*

| **Variable** |  | **Predictors Entered**  **Separately** | | | **Predictors Entered Jointly** | |
| --- | --- | --- | --- | --- | --- | --- |
|  |  | ***β*** | ***p*** | ***Pseudo-R^2^*** | ***β*** | ***p*** |
| *Demographic variables* | |  |  |  |  |  |
| Age | | .14 | .051 | .02 |  |  |
| Age^2^ | | .15 | .033 | .02 | .17 | .113 |
| % Female | | .00 | .928 | .03 | -.04 | .600 |
| % Not Non-Hispanic White | | -.11 | .109 | .01 | .05 | .554 |
| Education |  | -.08 | .253 | .01 | -.07 | .428 |
| Income | | .01 | .856 | .00 | .09 | .293 |
| % No full-time employment | | .04 | .564 | .00 | -.04 | .586 |
| % Not married | | .03 | .713 | .00 | -.04 | .611 |
| Political worldview | | -.19 | .006 | .11 | -.10 | .219 |
| *Individual difference measures* | |  |  |  |  |  |
| % Financial fraud victim | | -.06 | .418 | .01 | .04 | .585 |
| Bullshit receptivity | | -.26 | < .001 | .13 | .08 | .394 |
| Ad skepticism | | .31 | < .001 | .17 | .08 | .423 |
| Stress symptoms | | -.01 | .921 | .04 | .08 | .483 |
| Stress frequency | | .09 | .282 | .70 | .00 | .997 |
| Lack of premeditation | | -.08 | .235 | .02 | -.04 | .683 |
| Lack of perseverance | | .04 | .604 | .02 | -.05 | .518 |
| Sensation seeking | | -.03 | .658 | .03 | .08 | .357 |
| Positive urgency | | -.19 | .005 | .09 | .12 | .343 |
| Negative urgency | | -.15 | .031 | .02 | -.03 | .809 |
| *Solicitation measures* | |  |  |  |  |  |
| Action intentions | | -.58 | < .001 | .43 | -.53 | < .001 |
| Benefit rating | | -.58 | < .001 | .37 | -.05 | .716 |
| Perceived genuineness | | -.58 | < .001 | .36 | -.21 | .079 |
| Intercept | |  |  |  | .00 | < .001 |
| *Pseudo-R^2^* | |  | | | .98 | |
